# Irradiance Restoration Based Shadow Compensation Approach for High Resolution Multispectral Satellite Remote Sensing Images

**DOI:** 10.3390/s20216053

**Published:** 2020-10-24

**Authors:** Hongyin Han, Chengshan Han, Liang Huang, Taiji Lan, Xucheng Xue

**Affiliations:** 1 Changchun Institute of Optics, Fine Mechanics and Physics, Chinese Academy of Sciences, Changchun 130033, China; hanhongyin15@mails.ucas.edu.cn (H.H.); hancs@ciomp.ac.cn (C.H.); huangliang@ciomp.ac.cn (L.H.); lantaiji@ciomp.ac.cn (T.L.); 2 University of Chinese Academy of Sciences, Beijing 100049, China

**Keywords:** irradiance restoration, shadow compensation, WorldView-3, multispectral satellite remote sensing image

## Abstract

Numerous applications are hindered by shadows in high resolution satellite remote sensing images, like image classification, target recognition and change detection. In order to improve remote sensing image utilization, significant importance appears for restoring surface feature information under shadow regions. Problems inevitably occur for current shadow compensation methods in processing high resolution multispectral satellite remote sensing images, such as color distortion of compensated shadow and interference of non-shadow. In this study, to further settle these problems, we analyzed the surface irradiance of both shadow and non-shadow areas based on a satellite sensor imaging mechanism and radiative transfer theory, and finally develop an irradiance restoration based (IRB) shadow compensation approach under the assumption that the shadow area owns the same irradiance to the nearby non-shadow area containing the same type features. To validate the performance of the proposed IRB approach for shadow compensation, we tested numerous images of WorldView-2 and WorldView-3 acquired at different sites and times. We particularly evaluated the shadow compensation performance of the proposed IRB approach by qualitative visual sense comparison and quantitative assessment with two WorldView-3 test images of Tripoli, Libya. The resulting images automatically produced by our IRB method deliver a good visual sense and relatively low relative root mean square error (rRMSE) values. Experimental results show that the proposed IRB shadow compensation approach can not only compensate information of surface features in shadow areas both effectively and automatically, but can also well preserve information of objects in non-shadow regions for high resolution multispectral satellite remote sensing images.

## 1. Introduction

Optical satellite remote sensing images play an increasingly important role in a number of applications like building reconstruction, height evaluation, resource assessment, change detection and precision agriculture, along with the increasingly spatial resolution of optical satellite remote sensing images provided by high spatial resolution (HSR) satellites, such as QuickBird, WorldView-2, WorldView-3, and Jilin-1 [1,2,3,4,5,6,7,8]. However, shadows inevitably formed by clouds and land surface features interfere more seriously in these HSR optical satellite remote sensing images [9]. Notably, the shadow often weakens feature information due to the absence of direct light compared with the corresponding non-shadow [9,10,11]. Besides, significant information not only exists in most non-shadow regions, but also appears in shadow regions for applications like image classification, change detection, and target detection. Given this situation, proper correction of the shadow plays an important role for further applications of these valued HSR optical satellite remote sensing images. Therefore, shadows should be well pre-processed before additional applications of these HSR images.

Shadow is normally processed with two main steps: shadow detection (or extraction) and shadow compensation (or restoration). Researchers have devoted themselves to shadow detection and compensation [9,10,11,12,13,14,15,16,17,18,19,20]. In this paper, we make every reasonable effort to develop a relatively simple but effective automatic shadow compensation method. Numerous shadow compensation methods have been presented to alleviate the negative shadow interference of images. These methods can be mainly categorized into two types: the image enhancement (IE) based class and the imaging mechanism (IM) based class (also called the irradiance-based one).

Typical IE-based shadow compensation methods include statistical correction (like histogram matching, gamma correction, linear correlation correction (LCC) and gray stretching) [13,21,22,23], Retinex-based correction [24], and homomorphic filtering (HF) [25]. Suzuki et al. [26] presented a dynamic shadow compensation method, which could enhance the visibility of features under shadow regions in urban aerial images. This method compensated both the intensity and saturation per pixel of the shadow in accordance with the pre-calculated posterior probabilities. Wang et al. [27] compensated shadows in color aerial remote sensing images by applying different adjustment parameters over red (R), green (G), and blue (B) components, respectively. This shadow compensation method well improved the visibility of features in the shadow areas blocked by trees or buildings. Guo et al. [28] developed a simple shadow compensation algorithm for IKONOS images by mapping the gray values for pixels in both shadow and non-shadow regions and eliminating the visual difference by histogram matching. Sarabandi et al. [29] analyzed shadow compensation effects of histogram matching, gamma correction, and LCC for IKONOS and QuickBird images, and drew a conclusion that the LCC method performed better than histogram matching and gamma correction for these satellite remote sensing images. Su et al. [30] also provided a shadow compensation method by utilizing histogram matching in HIS color space rather than usually in RGB space. Chen et al. [31] compensated shadows based on the statistical relationship between shadow and non-shadow regions. In addition, Yamaziki et al. [14] and Liu et al. [16] developed a shadow compensation method with a piecewise linear equation based on the LCC method, which delivered an excellent performance over QuickBird images. As for the Retinex-based correction, related research mainly focused on the single scale Retinex (SSR) [32] and the multi-scale Retinex (MSR) [24]. Related research on shadow compensation also appeared in the frequency domain. For instance, Hao et al. [25] applied the HF technology on compensating shadows in SPOT remote sensing images, which offered a novel thought.

Additionally, the IM-based methods have attracted more attention in recent years. Guo et al. [15] developed a new imaging model based on the radiative transfer theory to compensate for the lost information in shadow areas, where shadow could be considerably suppressed. Ye et al. [33] explored a light source color rate (LSCR) based shadow compensation method for aerial images with the Shades of Gray algorithm [34], in which the uncertainty caused by parameters of the Minkowski norm was further analyzed. Wen et al. [9] also proposed a shadow compensation approach for various scales remote sensing images based on the reflectance equality relationship (RER) reducing the number of parameters and the corresponding error propagated by these parameters although the shadow parameter *f* was computed manually. Lin et al. [35] and Silva et al. [11] also presented the direct and environment light based method (DELM) for shadow compensation purpose based on the illumination distinction between shadow and non-shadow areas.

Though numerous shadow detection methods have been developed, problems inevitably occur for current shadow compensation methods in processing high resolution multispectral satellite remote sensing images, such as color distortion of compensated shadow and interference of non-shadow. In this paper, we proposed an irradiance restoration based (IRB) shadow compensation approach for HSR optical multispectral satellite remote sensing images by simplifying the isotropic reflected irradiance based on a single angle, which was developed under the assumption that the shadow area owns the same irradiance to the nearby non-shadow area containing the same type features. For testing the shadow compensation performance of our proposed IRB approach, we carried out comparative experiments over test images of WorldView-2 and WorldView-3 acquired at different sites and times. Particularly, we evaluated the shadow compensation performance of the proposed IRB approach both qualitatively and quantitatively against several shadow compensation methods (i.e., LCC [16,21], LSCR [33], MSR [24,36], HF [25] and DELM [11,37]) with two WorldView-3 test images of Tripoli, Libya.

The rest proceeds as in the following. The proposed IRB shadow compensation approach is described in detail in Section 2. Performance evaluation is conducted both qualitatively and quantitatively with comparative experiments in Section 3. Discussion of influential elements is presented in Section 4. Conclusions are finally drawn in Section 5.

## 2. Method

### 2.1. The Derivation of the Irradiance Restoration Based (IRB) Approach

**Figure 1:**
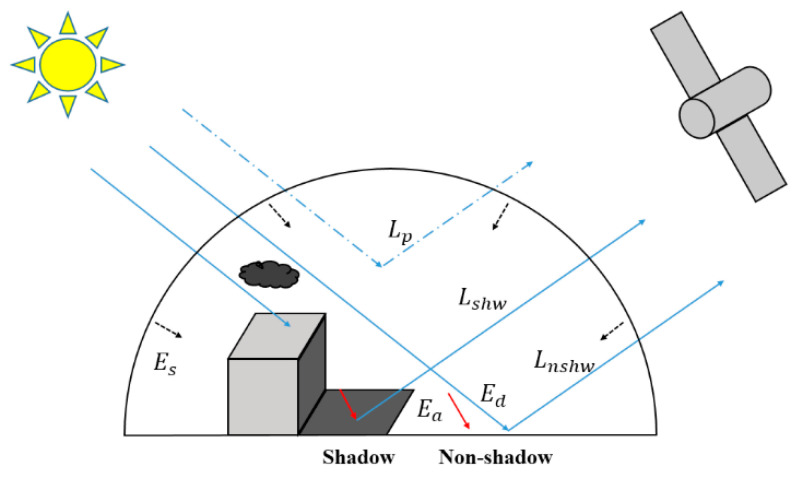
Illustration of the light transfer process in non-shadow and nearby shadow regions [9].

The sun is usually regarded as the unique light source in optical satellite remote sensing imaging fields. Figure 1 illustrates the light transfer process in both non-shadow and nearby shadow regions for the optical satellite sensor [9].

Specifically, the surface irradiance ($E_{nshw}$) of the non-shadow aera includes three parts: direct irradiance ($E_{d}$), scattered irradiance ($E_{s}$) (also named diffuse irradiance), and ambient (or reflected) irradiance ($E_{a}$). However, the surface irradiance ($E_{shw}$) of the shadow area mainly consists of $E_{s}$ and $E_{a}$, because $E_{d}$ is often inevitably occluded by clouds and surface features for the shadow area. The surface irradiance compositions of the non-shadow area and the shadow area are expressed in the following equations [25,35,38]:

(1) $$E_{nshw}=E_{d}+E_{s}+E_{a}$$

(2) $$E_{shw}=E_{s}+E_{a}$$

Generally, the radiant existence ($M_{nshw}$) of the Earth’s surface in the non-shadow area is expressed with the corresponding $E_{nshw}$:

(3) $$M_{nshw}=\rho_{nshw}E_{nshw}$$

where $\rho_{nshw}$ is the surface reflectance of the non-shadow area.

In addition, the radiance from the Earth’s surface of the non-shadow area ($L_{nshw}^{earth}$) can be expressed with the corresponding radiant existence ($M_{nshw}$) of the Earth surface in the non-shadow area:

(4) $$L_{nshw}^{earth}=\frac{M_{nshw}}{\pi }$$

where π is the solid angle.

Therefore, $L_{nshw}^{earth}$ can be directly described with the corresponding $E_{nshw}$ by substituting Equation (3) for $M_{nshw}$ in Equation (4), as follows [39]:

(5) $$L_{nshw}^{earth}=\frac{\rho_{nshw}}{\pi }E_{nshw}$$

Our purpose is to restore information of shadow regions in the acquired images. Therefore, we mainly concentrate on the light propagation path from the Earth surface to the satellite sensor. In the light propagation process, atmospheric influence should be taken into consideration, because the radiance of land-surface features propagates through the atmosphere before arriving at the satellite sensor. The atmospheric influence mainly results in an attenuated radiance from the Earth surface, and a path radiance ($L_{p}$) that is directly reflected to the satellite sensor by the atmosphere, but does not arrive at the Earth surface [40]. Hence, the total radiance of the non-shadow area arriving at the satellite sensor ($L_{nshw}^{sensor}$) can be expressed with the attenuated $L_{nshw}^{earth}$ and $L_{p}$:

(6) $$L_{nshw}^{sensor}=\tau L_{nshw}^{earth}+L_{p}$$

where τ is the atmospheric transmittance from the Earth surface to the satellite sensor.

Here, the image radiance of the non-shadow area is calculated in accordance with Equations (1) and (5) as the following equation:

(7) $$\begin{array}{ll} L_{nshw}^{sensor} & =\frac{\tau \rho_{nshw}}{\pi }E_{nshw}+L_{p}\\ & =\frac{\tau \rho_{nshw}}{\pi }\left(E_{d}+E_{s}+E_{a}\right)+L_{p} \end{array}$$

Similarly, the image radiance of the shadow area is also expressed in a similar way:

(8) $$\begin{array}{ll} L_{shw}^{sensor} & =\frac{\tau \rho_{shw}}{\pi }E_{shw}+L_{p}\\ & =\frac{\tau \rho_{shw}}{\pi }\left(E_{s}+E_{a}\right)+L_{p} \end{array}$$

where $\rho_{shw}$ is the surface reflectance of the shadow area.

Thus, image radiance can be described in a general way combining Equations (7) and (8), as follows [11]:

(9) $$L=\frac{\tau \rho }{\pi }\left(kE_{d}+E_{s}+E_{a}\right)+L_{p}$$

where ρ is the surface reflectance, and k is the shadow approximation parameter, in which k=0 refers to shadow and k=1 denotes non-shadow.

Additionally, the absence of $E_{d}$ in the shadow area contributes to the difference between non-shadow and shadow areas. For retrieving information of the shadow area containing the same type features to the nearby non-shadow area, an assumption can be arranged in which the same surface irradiance of the non-shadow area is owned by the nearby shadow area. Namely, the shadow area receives $E_{s}$, $E_{a}$, and a hypothesized direct irradiance ($E_{d}^{hyp}$) under the assumption above. In this case, the corresponding hypothesized radiance of the shadow area ($L_{shw}^{hyp}$) can be considered as the compensated radiance, which is received by the satellite sensor hypothetically but not actually, as shown in Equation (10).

(10) $$\begin{array}{ll} L_{shw}^{hyp} & =\frac{\tau \rho_{shw}}{\pi }\left(E_{d}^{hyp}+E_{s}+E_{a}\right)+L_{p}\\ & =\frac{\tau \rho_{shw}}{\pi }\frac{\left(kE_{d}+E_{s}+E_{a}\right)\left(E_{d}^{hyp}+E_{s}+E_{a}\right)}{\left(kE_{d}+E_{s}+E_{a}\right)}+L_{p}\\ & =\left(L-L_{p}\right)\frac{\left(E_{d}^{hyp}+E_{s}+E_{a}\right)}{\left(kE_{d}+E_{s}+E_{a}\right)}+L_{p} \end{array}$$

In the shadow compensation process, because $E_{d}^{hyp}$ is developed under the assumption that the shadow area contains the same type features as the nearby non-shadow area, $E_{d}^{hyp}$ can be regarded approximately equal to $E_{d}$ in the corresponding nearby non-shadow area (i.e., $E_{d}^{hyp}=E_{d}$). Therefore, Equation (10) can be rewritten as in the following:

(11) $$\begin{array}{ll} L_{shw}^{hyp} & =\left(L-L_{p}\right)\frac{\left(\frac{E_{d}^{hyp}}{E_{s}+E_{a}}+1\right)}{\left(k\frac{E_{d}}{E_{s}+E_{a}}+1\right)}+L_{p}\\ & =\left(L-L_{p}\right)\frac{\left(r+1\right)}{\left(kr+1\right)}+L_{p} \end{array}$$

where r is the irradiance coefficient defined by Equation (12).

(12) $$r=\frac{E_{d}^{hyp}}{E_{s}+E_{a}}=\frac{E_{d}}{E_{s}+E_{a}}$$

In order to calculate r with image radiance, $L_{p}$ is first moved from the right-hand side to the left-hand side in both Equations (7) and (8), then items of Equation (8) are divided by the corresponding items of Equation (7), as shown in Equation (13):

(13) $$\frac{L_{shw}^{sensor}-L_{p}}{L_{nshw}^{sensor}-L_{p}}=\frac{\rho_{shw}\left(E_{s}+E_{a}\right)}{\rho_{nshw}\left(E_{d}+E_{s}+E_{a}\right)}$$

In addition, the surface reflectance of the non-shadow area is reasonably equal to that of the nearby shadow area containing the same type features (i.e., $\rho_{nshw}=\rho_{shw}$) [9]. Thus, Equation (13) can be further rewritten as Equation (14):

(14) $$\frac{L_{shw}^{sensor}-L_{p}}{L_{nshw}^{sensor}-L_{p}}=\frac{E_{s}+E_{a}}{E_{d}+E_{s}+E_{a}}=\frac{1}{r+1}$$

Hence, the irradiance coefficient r can be obtained in the image radiance from with the following equation:

(15) $$r=\frac{L_{nshw}^{sensor}-L_{shw}^{sensor}}{L_{shw}^{sensor}-L_{p}}$$

For clarity, the compensated image radiance of the shadow area ($L_{shw}^{comp}$) can be represented by the following equation:

(16) $$L_{shw}^{comp}=\left(L-L_{p}\right)\frac{\left(r+1\right)}{\left(kr+1\right)}+L_{p}$$

Note that Equation (16) only needs to estimate $L_{p}$ avoiding the uncertainty of the estimation of parameters τ and ρ. Thus, the solution of Equation (16) is promising. 

For pixels of both shadow and non-shadow areas in the target image, Equation (16) can be further expressed as follows:

(17) $$L_{shw}^{comp}=\left\{ \begin{array}{ll} L & k=1\\ L+r\left(L-L_{p}\right) & k=0 \end{array}\right.$$

### 2.2. Workflow of the IRB Approach

**Figure 2:**
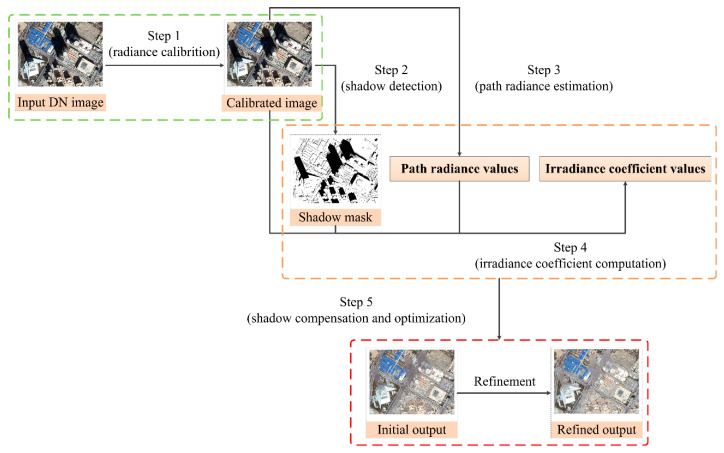
Workflow chart for the IRB approach.

In our study, the target image is first divided into shadow and non-shadow regions with the logarithmic shadow index (LSI) shadow detection method and further refined with a certain manual operation. Consequently, shadow regions are compensated with the proposed IRB method pixel-by-pixel and non-shadow regions are left unchanged. The developed IRB approach is mainly accomplished in five steps described as in the following, as shown in Figure 2.

- Step 1: Radiance calibration

Optical satellite remote sensing images are usually saved in a digital number (DN) value form. For instance, the Worldview-3 data is saved in the 11-bit DN value form [41]. Radiance calibration is often applied prior to shadow correction, in which the image DN value is converted into radiance pixel by pixel with the image metadata in the acquired image files through a certain calibration algorithm or directly over a professional software. In this study, the radiance calibration process is carried out with the radiometric calibration module in the ENVI 5.2.

- Step 2: Shadow detection

In the shadow correction process, shadow detection is a pre-process prior to shadow compensation, which extracts shadow regions in the target image and classifies the target image into non-shadow and shadow parts [42,43,44,45]. In this step, the LSI shadow detection method [46] is first utilized to produce the shadow mask image. Problems of non-shadow misclassification and shadow omission are still inevitable for the current shadow detection process. Consequently, we manually refine the shadow detection result of the LSI shadow detection method to further alleviate shadow detection problems.

- Step 3: Path radiance estimation

The path radiance $L_{p}$ is an important element for the whole shadow compensation process, which can be estimated with various algorithms, like the dark object subtraction (DOS), the histogram method, and the improved dark object subtraction (IDOS) [47,48]. In this study, we estimate the $L_{p}$ value per band of the target image with the IDOS algorithm.

- Step 4: Irradiance coefficient computation

As shown in Equation (15), the irradiance coefficient r is derived with $L_{nshw}^{sensor}$, $L_{shw}^{sensor}$ and $L_{p}$. As mentioned in Step 3 above, $L_{p}$ values are estimated with the IDOS algorithm. In this step, we compute $L_{nshw}^{sensor}$ and $L_{shw}^{sensor}$ with the Minkowski norm on the basis of the color constancy theory [33,34], respectively, as shown in Equation (18):

(18) $$L_{est}=a{\left[\frac{\sum_{x=1}^{M}\sum_{y=1}^{N}{\left(f(x,y)\right)}^{p}}{M\times N}\right]}^{\frac{1}{p}}$$

where a is the gain factor, p is an adjustable parameter, f(x,y) is the radiance value of the target image with the coordination (x,y), and M×N refers to the amount of pixels in either the non-shadow area or the shadow area of the target image.

- Step 5: Shadow compensation and optimization

The shadow compensation task is initially accomplished with the shadow detection result (Step 2), $L_{p}$ (Step 3) and r (Step 4) in accordance with the solution of Equation (17). 

For pixels belonging to the shadow area (i.e., k=0), refining parameters α and β are additionally applied to optimize the compensation result by the solution of Equation (17). The optimized compensated radiance $L_{shw}^{opt}$ is shown with the following equation:

(19) $$L_{shw}^{opt}=\left\{ \begin{array}{ll} L & k=1\\ \alpha L+\beta r\left(L-L_{p}\right) & k=0 \end{array}\right.$$

where α and β are refining parameters. 

As shown in Equation (19), refining parameters α and β are additionally applied to optimize the shadow compensation result by the initial solution of Equation (17), which contributes to the refined resulting image after shadow compensation. Particularly, parameter α is mainly employed to optimize the impact of the original shadow on the compensation result, and parameter β is utilized to refine the influence of the path radiance and the irradiance coefficient on the compensation result. However, it is unrealistic to form an explicit mathematical model for the refining parameters used in the image interpretation of high resolution remote sensing images, because complex and diverse ground features are usually caught in these images. Therefore, we refer to the experimental solution with similar optimization for defining the refining parameters for shadow detection of high resolution remote sensing images by Zhang et al. [49]. We finally decide to choose the experimental solution to defining the refining parameters α and β. As for the restriction of the refining parameters α and β, the additional experimental results reveals that the restriction of α+β is both simple and valid for optimizing the compensation result, although there are many ways to restrict the refining parameters α and β. With these considerations, we further explore the restriction of α+β by ranging α+β from 1 to 6 in experiments with both the quantitative measurement, relative root mean square error (rRMSE), and the visual sense of compensation results. We finally determine to restrict the refining parameters α and β with α+β=3. Subsequently, under the restriction of α+β=3, we traverse the refining parameters α and β in the interval of 0.1 from 0.1 to 3, and define values of α and β. A related discussion and analysis on the parameter sensitivity of compensation results are provided in additional experiments in Section 4. 

## 3. Performance Evaluation

### 3.1. Test Images

**Figure 3:**
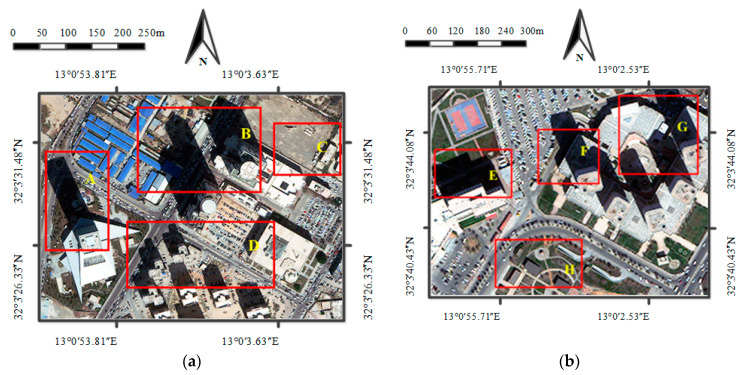
Two test images from WorldView-3 images of Tripoli, Lebanon. (**a**) Tripoli-1. (**b**) Tripoli-2.

The proposed IRB shadow compensation approach was accomplished on a DELL personal computer with 64-bit Windows 7 operation system equipped with a 3.2 GHz CPU and a 4 GB RAM. To verify the shadow compensation performance of the proposed IRB approach, we run comparative experiments with many images from WorldView-3 of Tripoli, Libya, and Rio de Janeiro, Brazil, and WorldView-2 of Washington DC, USA, which are respectively called WV3-Tripoli, WV3-Rio, and WV2-WDC. The corresponding discussion is provided in the next section (Section 4: Discussion). Particularly, qualitative and quantitative assessments are performed in this section to evaluate the shadow compensation performance of the proposed IRB approach against several shadow compensation methods (i.e., LCC, LSCR, MSR, HF, and DELM) with two test images from WorldView-3 of Tripoli, Libya [41], as shown in Figure 3a,b (respectively called Tripoli-1 and Tripoli-2). Specifically, the test image Tripoli-1 in Figure 3a is a 400 × 300 pixel image, which covers typical ground objects, such as shadow, various scale urban buildings, asphalt roads, bare land, and grass. The test image Tripoli-2 in Figure 3b is a 260 × 195 pixel image mainly consisting of shadow, buildings, asphalt roads, grass, playground, and parks. 

### 3.2. Qualititave Evaluation

For evaluating shadow compensation results, the subjective visual sense evaluation method intuitively reflects the compensation effect of specific regions in target images and is conducive to analyzing the performance among various shadow compensation methods for a specific shadow [14,16]. In this part, the shadow compensation effect is first analyzed for test images Tripoli-1 and Tripoli-2 from the perspective of qualitative visual sense comparison. Shadow compensation results of test images by the proposed IRB shadow compensation approach are respectively discussed in terms of R, G and B components. Then, a comprehensive visual sense comparison is also made between shadow compensation results by various shadow compensation algorithms (i.e., IRB, LCC, LSCR, MSR, HF, and DELM). 

**Figure 4:**
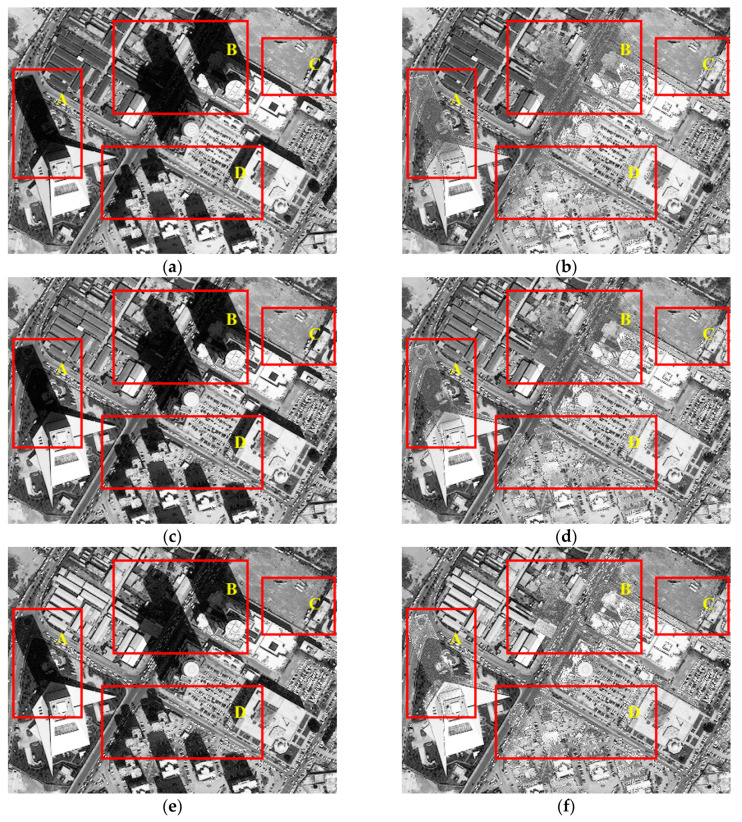
Resulting images of the test image Tripoli-1 compensated before and after by the IRB shadow compensation algorithm in terms of red (R), green (G), and blue (B) components. (**a**) R before shadow compensation. (**b**) R after shadow compensation. (**c**) B before shadow compensation. (**d**) B after shadow compensation. (**e**) G before shadow compensation. (**f**) G after shadow compensation.

Figure 4 lists the resulting images of the test image Tripoli-1 compensated before and after by the presented IRB approach in terms of R, G, and B components. As shown in Figure 4b, significant improvement in the gray value of shadow regions is achieved in the R component of the resulting image compared with the original one in Figure 3a. In particular, most areas with relatively severe shadow effect in Figure 4a are well compensated in Figure 4b, such as areas A-D. Moreover, information of features in shadow regions in Figure 4a is mostly restored in Figure 4b compared with nearby similar ground features, which can be obviously seen by comparing small buildings in region A and bare ground and asphalt roads in region B in both Figure 4a,b. Therefore, the R component of Tripoli-1 processed by IRB shows a significant improvement in visual sense opposed with the original one. Similarly, shadow regions of G and B components in Figure 4d,f also achieve obvious visual sense improvement, which is consistent with the compensation result of R component in Figure 4b, when compared with those in Figure 4c,e. Consequently, not only is obvious improvement in visual sense acquired by the IRB shadow compensation approach for the test image Tripoli-1, but good consistency is also shown among the compensation results of R, G, and B components. 

**Figure 5:**
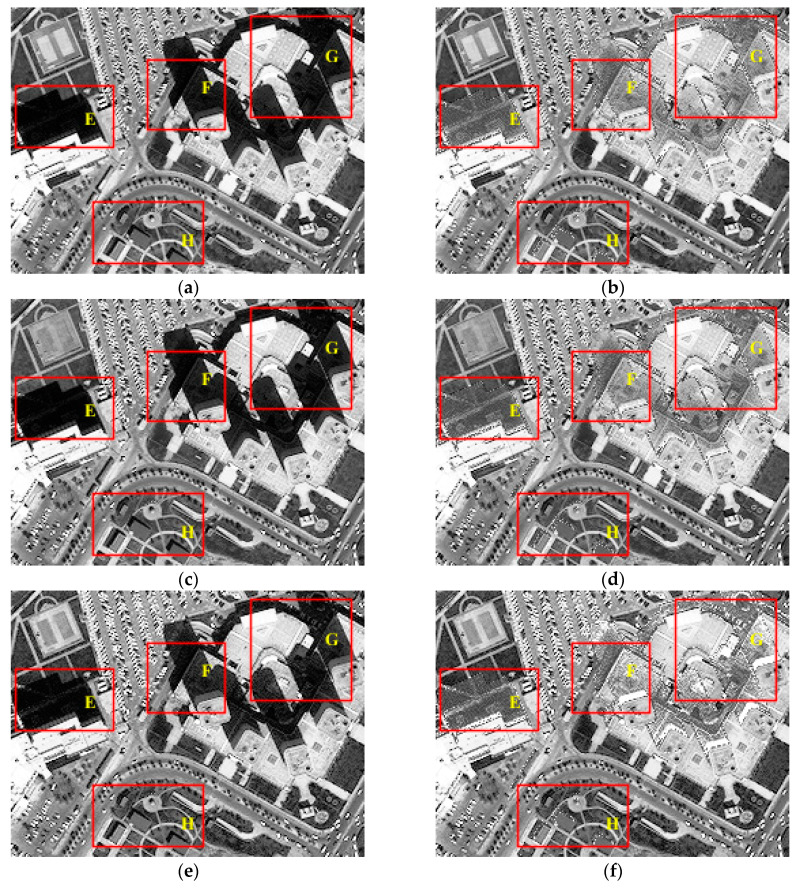
Resulting images of the test image Tripoli-2 compensated before and after by the IRB shadow compensation algorithm in terms of R, G and B components. (**a**) R before shadow compensation. (**b**) R after shadow compensation. (**c**) B before shadow compensation. (**d**) B after shadow compensation. (**e**) G before shadow compensation. (**f**) G after shadow compensation.

Figure 5 also presents R, G, and B components of test image Tripoli-2 compensated before and after by the proposed IRB shadow compensation method. As shown in Figure 5b, obvious improvement in brightness of shadow regions is observed intuitively in the R component of Tripoli-2 compensated by the IRB method on comparing shadow regions in the original R component of Tripoli-2 shown in Figure 5a and the corresponding ones in the compensated R component shown in Figure 5b. For instance, several ground objects covered by shadow in regions E-H are almost completely revealed in the compensated R component of Tripoli-2 seen in Figure 5b. Specifically, by comparing ground features under shadow in regions E-H of both Figure 5a,b, the information of typical ground targets is clearly restored by the IRB method in Figure 5b, like grass under building shadow in region E, asphalt roads in region F, tops of buildings in region G, and grass under small shadow in region H, which are mostly covered by shadow in Figure 5a. Therefore, it can be observed that the R component of Tripoli-2 is well compensated by the proposed IRB method, and the visual sense of the compensated R component has been significantly improved compared with the original one. Analogous to the compensation result of the R component of Tripoli-2, G and B components also acquire a dramatical improvement in terms of the gray value of shadow regions, as shown in Figure 5d,f. Moreover, the compensation results of R, G, and B components are relatively consistent. Hence, not only is meaningful improvement of the visual sense achieved by the IRB shadow compensation approach, but strong consistency is also shown in compensation results in terms of R, G, and B components of Tripoli-2.

In addition to visual sense comparison regarding compensation results by the IRB shadow compensation approach in terms of R, G, and B components of test images Tripoli-1 and Tripoli-2, related analysis is also carried out to compare compensation results by the IRB approach and several shadow compensation methods (i.e., LCC, LSCR, MSR, HF, and DELM). 

**Figure 6:**
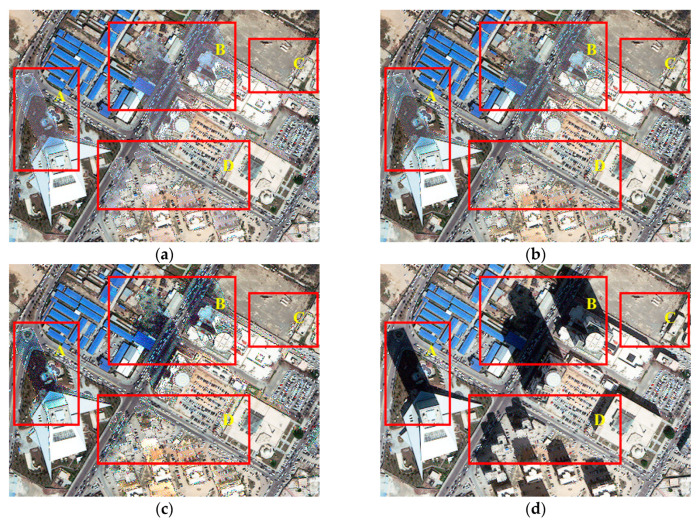

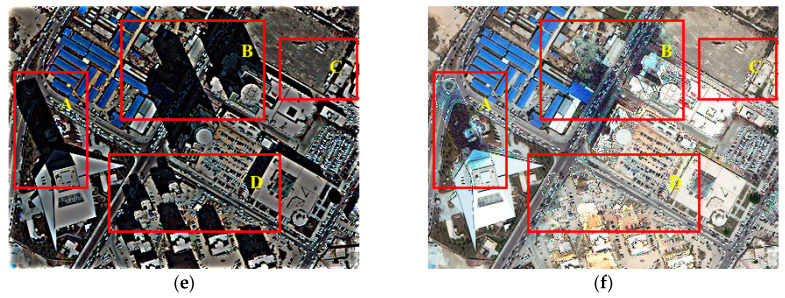
Shadow compensation results of various shadow compensation algorithms for the test image Tripoli-1. (**a**) The irradiance restoration based (IRB) method. (**b**) The linear correlation correction (LCC) method [16,21]. (**c**) The light source color rate (LSCR) method [33]. (**d**) The multi-scale Retinex (MSR) method [24,36]. (**e**) The homomorphic filtering (HF) method [25]. (**f**) The direct and environment light based method (DELM) [11,37].

Figure 6 illustrates the shadow compensation results of the test image Tripoli-1 by the IRB approach and several shadow compensation methods (i.e., LCC, LSCR, MSR, HF, and DELM). As presented in Figure 6a, obviously, the resulting image by the IRB method reveals more details about ground targets under shadow and significant improvement is also shown in a visual sense by comparing the shadow compensation result in Figure 6a with the original test image Tripoli-1 in Figure 3a. A similar color effect of shadow regions is also achieved in the compensation result to that of the adjacent similar objects, such as small buildings and grass in region A, blue houses in region B, and nearby shadow of buildings in regions C and D shown in Figure 6a. As illustrated in Figure 6b, the shadow compensation result by LCC also improves the visibility of objects in the shadow regions. Similarly, the shadow compensation result by LSCR and DELM in Figure 6c,f also shows great improvement of shadow areas in visual sense, even though the improvement effect is not as obvious as the results by IRB and LCC listed in Figure 6a,b. However, the shadow effect is still obvious in the resulting image by the MSR shadow compensation method shown in Figure 6, like almost similar shadow regions A–D appearing in Figure 3a. Additionally, color distortion and non-shadow interference occur obviously in the resulting image by HF, as shown in Figure 6e. Generally speaking, the proposed IRB algorithm well restores the information of objects in shadow regions of the test image Tripoli-1, and achieves a relatively good visual sense compared with the comparative shadow compensation methods.

**Figure 7:**
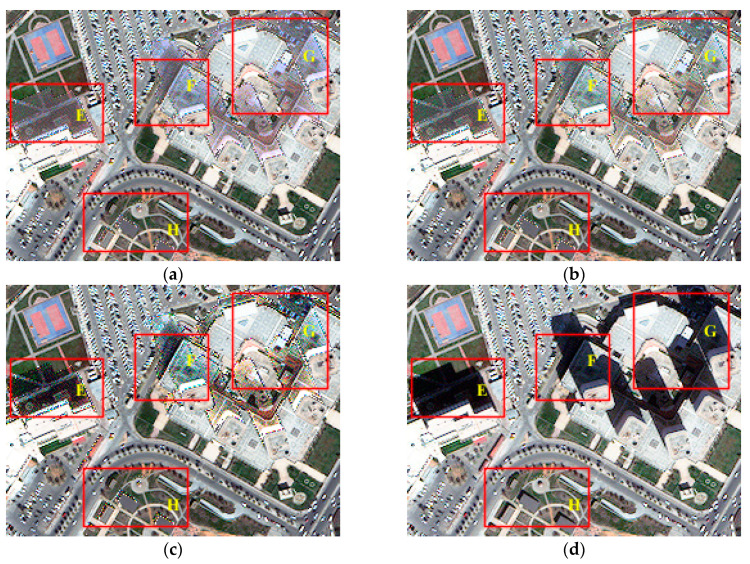

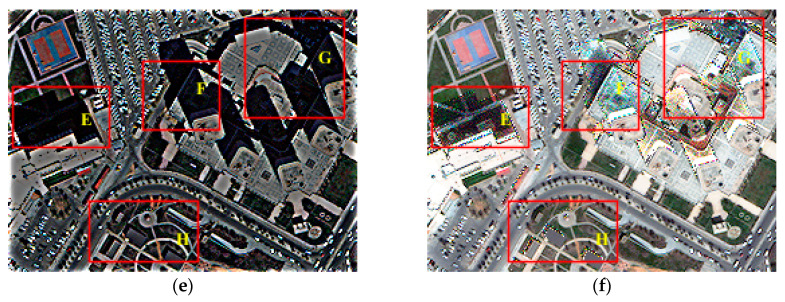
Shadow compensation results of various shadow compensation algorithms for the test image Tripoli-2. (**a**) IRB. (**b**) LCC. (**c**) LSCR. (**d**) MSR. (**e**) HF. (**f**) DELM.

Additionally, Figure 7 also depicts shadow compensation results of the test image Tripoli-2 by the IRB approach and several shadow compensation methods (i.e., LCC, LSCR, MSR, HF, and DELM). Similar to the discussion of Tripoli-1, IRB, LCC, LSCR, and DELM also perform well in shadow compensation of Tripoli-2 and achieve a dramatical improvement of shadow regions in visual sense. For instance, as shown in Figure 7a–c and f, most ground objects under shadow are well restored, like grass under building shadow in region E, asphalt roads in region F, tops of buildings in region G, and grass under small shadow in region H. However, as presented in Figure 7d, shadow still exists obviously in the resulting image by MSR, like large building shadow in regions E, F and G, and small house shadow in region H. In addition, color distortion and non-shadow interference still appear in the resulting image by HF shown in Figure 7e. In general, obvious improvement of shadow in the test image Tripoli-2 in terms of visual sense is achieved in resulting images by IRB, LCC, LSCR, and DELM. 

All in all, by comprehensively comparing shadow compensation results of test images Tripoli-1 and Tripoli-2 by IRB and several shadow compensation methods (i.e., LCC, LSCR, MSR, HF, and DELM), it can be seen that the proposed IRB shadow compensation approach can well restore features of shadow regions and achieve a relatively good overall visual sense. 

### 3.3. Quantitative Assessment

In addition to the qualitative evaluation above, the quantitative assessment method is also an effective assessment way. Meanwhile, the quantitative assessment method is also used to evaluate the performance of shadow compensation methods for further quantitatively assessing the shadow compensation results. The root mean square error (RMSE) is a widely used indicator for quantitative assessment [9]. However, considering the difference of the absolute RMSE values for shadow compensation results in terms of R, G, and B components, the relative RMSE (rRMSE) is more reasonable instead of the absolute RMSE to assess shadow compensation results in terms of R, G, and B components. Accordingly, the rRMSE is defined by the following equation [9]:

(20) $$rRMSE=\sqrt{\frac{1}{M\times N}\times \sum_{x=1}^{M}\sum_{y=1}^{N}{\left[\frac{f_{ref}\left(x,y\right)-f_{result}\left(x,y\right)}{f_{ref}\left(x,y\right)}\right]}^{2}}\times 100\%$$

where $f_{result}\left(x,y\right)$ and $f_{ref}(x,y)$ are radiance values of the shadow compensation resulting image and the corresponding reference image with the coordination (x,y), and M×N refers to the amount of pixels. 

**Figure 8:**
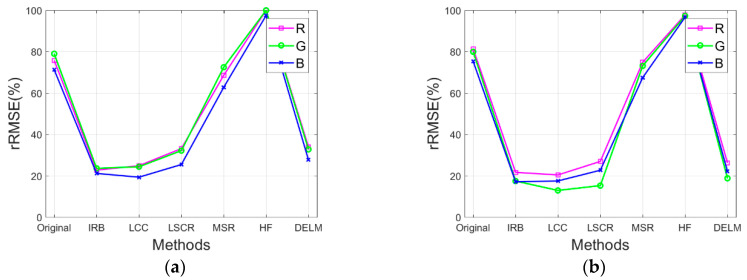
rRMSE of shadow compensation results for test images compensated before and after by various shadow compensation algorithms in terms of R, G, and B components. (**a**) Tripoli-1. (**b**) Tripoli-2.

Specifically, the performance of the IRB approach is evaluated by comparing rRMSE of the resulting image against the rRMSE of the original image and the rRMSE of resulting images by several shadow compensation methods (i.e., LCC, LSCR, MSR, HF, and DELM), where a smaller rRMSE value delivers a better shadow compensation performance. In the following section, rRMSE is mainly utilized to quantify the performance of the proposed IRB shadow compensation approach for test images Tripoli-1 and Tripoli-2 in terms of R, G, and B components. Figure 8a,b depict rRMSE of shadow compensation results for test images compensated before and after by various shadow compensation algorithms (i.e., IRB, LCC, LSCR, MSR, HF, and DELM) in terms of R, G, and B components, respectively. As shown in Figure 8a,b, obviously, rRMSE values of resulting images by MSR and HF are relatively high, which shows the poor shadow compensation ability of MSR and HF. However, relatively small rRMSE values of resulting images are acquired by IRB and LCC compared with the rRMSE of the original images for test images Tripoli-1 and Tripoli-2 in terms of R, G, and B components, which reveals the good shadow compensation performance of IRB and LCC. Similarly, small rRMSE values of resulting images are also achieved by LCSR and DELM compared with the rRMSE of the original images. Since the rRMSE values of resulting images by IRB and LCC are smaller than those by LCSR and DELM, IRB and LCC can deliver a better shadow compensation performance. 

**Figure 9:**
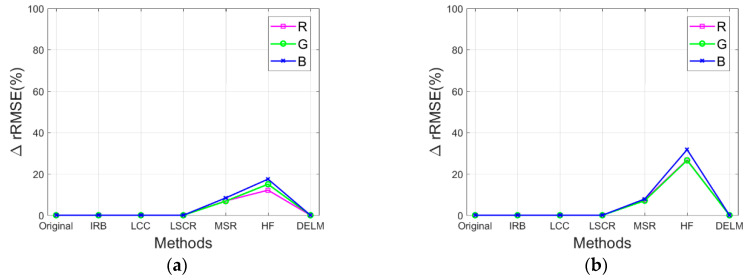
∆rRMSE between non-shadow regions in the original image and those in the shadow compensation resulting images by various shadow compensation algorithms for test images in terms of R, G, and B components. (**a**) Tripoli-1. (**b**) Tripoli-2.

Additionally, Figure 9a,b illustrate ∆rRMSE between non-shadow regions in the original image and those in the shadow compensation resulting images by various shadow compensation algorithms for test images in terms of R, G, and B components. As depicted in Figure 9a,b, ∆RMSE values are more than zero for resulting images by MSR and HF, which reveals that feature information in non-shadow regions are corrupted to some extent. However, ∆RMSE values are zero or approximately zero for resulting images by IRB, LCC, LSCR, and DELM, which delivers that IRB, LCC, LSCR, and DELM can well preserve feature information in non-shadow regions, while compensating for shadow. 

Generally speaking, the proposed IRB method not only further alleviates the color distortion problem compared with typical image enhancement based shadow compensation methods (like MSR and HF), but also acquires a relatively good visual sense similar to that by LCC. Besides, the relatively small rRMSE and zero or approximately zero ∆RMSE also reveal the relatively good performance of the proposed IRB method for shadow compensation and non-shadow preservation. Better agreement is also shown for R,G, and B components of results in terms of both visual sense and quantitative measurement (rRMSE) by the proposed IRB method against the compared methods. 

## 4. Discussion

The presented IRB algorithm achieves a good shadow compensation performance in the comparative experiments above with test images Tripoli-1 and Tripoli-2. However, as described in the workflow of the proposed IRB algorithm in Section 2, uncertainties may appear in Step 3 (path radiance estimation) and Step 4 (irradiance coefficient computation). Meanwhile, in Step 5 (shadow compensation), the shadow compensation result is also sensitive to refining parameters α and β. In this section, a corresponding discussion is given to evaluate the effect of these elements. Thus, additional experiments are performed to discuss potential influence elements of the IRB approach on the shadow compensation results with test images Tripoli-1 and Tripoli-2.

### 4.1. Influence Analysis of Path Radiance Estimation

The IDOS algorithm is utilized to estimate the path radiance value for each band of the target image. The relative scattering model and the starting haze value (SHV) band, which are used in the estimation of the path radiance with the IDOS algorithm, usually maintain the estimation result. Available bands of WorldView-3 [41] and regular five relative scattering models [47] are listed in Table 1 and Table 2, respectively. Because the test image is captured under sunny and cloudless atmospheric conditions, the corresponding atmospheric condition can be regarded as very clear. Namely, the $\lambda^{-4}$ relative scattering model [47] is applied during the estimation of path radiance. Hence, properly selecting the SHV band becomes the main influential element in the path radiance estimation.

**Figure 10:**
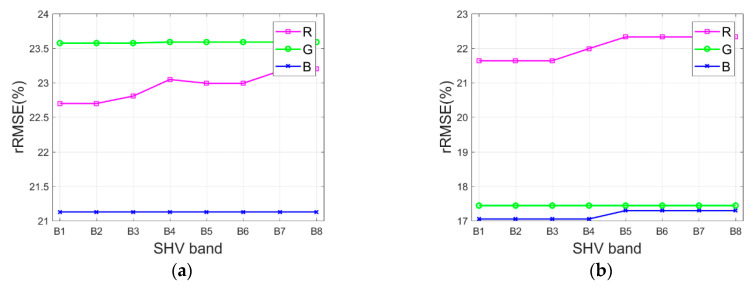
rRMSE of shadow compensation results with various starting haze value (SHV) bands for test images. (**a**) Tripoli-1. (**b**) Tripoli-2.

Consequently, in order to study the influence of the path radiance estimation on the shadow compensation results by the IRB algorithm, the available bands of test images (i.e., B1, B2, B3, B4, B5, B6, B7, and B8) are respectively set as the SHV band in the additional experiments with test images Tripoli-1 and Tripoli-2. Figure 10a,b illustrate the rRMSE of shadow compensation results with various SHV bands for test images Tripoli-1 and Tripoli-2 in terms of R, G, and B components, respectively. 

As illustrated in Figure 10a,b, it can be observed that the SHV band slightly impacts the shadow compensation performance of the IRB algorithm. Specifically, when B1, B2, or B3 is set as the SHV band for the IRB algorithm, relatively lower rRMSE values of R, G, and B components are almost acquired at the same time for test images Tripoli-1 and Tripoli-2, which shows that a better shadow compensation effect is achieved for test images Tripoli-1 and Tripoli-2 with B1, B2, or B3 as the SHV band. Accordingly, in this study, B2 is finally utilized as the optimized SHV band for test images.

### 4.2. Influence Analysis of Irradiance Coefficient Computation 

**Figure 11:**
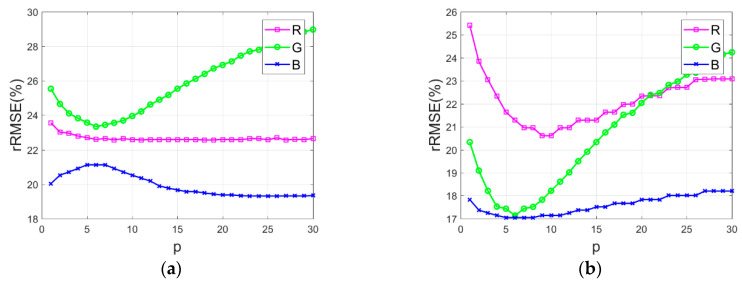
rRMSE of shadow compensation results with various *p* values for test images. (**a**) Tripoli-1. (**b**) Tripoli-2.

The Minkowski norm is utilized in the irradiance coefficient computation together with the path radiance. As for the calculation of the Minkowski norm, related studies [33,34] noted that uncertainties of shadow correction effect in natural and aerial images are mainly sensitive to the parameter *p*. In order to further explore the influence of the parameter *p* on the shadow compensation effect of HSR optical satellite remote sensing images, the parameter *p* is respectively set as from 1 to 30 with an integral interval of 1 in the additional experiments with test images Tripoli-1 and Tripoli-2. Figure 11a,b respectively present the rRMSE of shadow compensation results with various *p* values for test images Tripoli-1 and Tripoli-2 in terms of R, G, and B components. 

As can be observed in Figure 11a,b, obviously, relatively lower rRMSE values are acquired for R, G, and B components with *p* value of 4, 5, 6, or 7 for test images Tripoli-1 and Tripoli-2. Additionally, the rRMSE curves in Figure 11a,b show an increasing trend for test images Tripoli-1 and Triploli-2 with the *p* value from 1 to 30. Consequently, in this study, we handle test images with parameter *p* value of 5. 

### 4.3. Sensitivity Analysis of Refining Parameters

**Figure 12:**
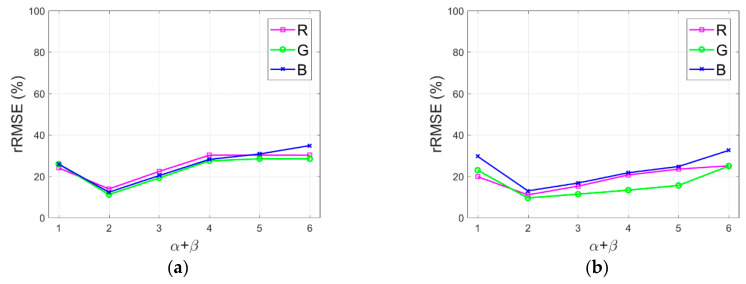
rRMSE of shadow compensation results with various α+β values for test images. (**a**) Tripoli-1. (**b**) Tripoli-2.

In this study, the initially derived shadow compensation solution is further optimized with parameters α and β. According to the optimized solution given in Equation (19), the resulting images are sensitive to refining parameters α and β. In particular, the refining parameter α optimizes the partial impact of the original shadow on the compensation results, and β mainly affects the influence of both the path radiance and irradiance coefficient on the compensation results. In this study, the relationship of α and β is roughly restricted with α+β. Subsequently, the influence of different α+β is analyzed by compensating test images with various α+β ranging from 1 to 6. Figure 12a,b illustrate rRMSE of shadow compensation results with various α+β values for test images Tripoli-1 and Tripoli-2 in terms of R, G, and B components. As presented in Figure 12a,b, relatively low rRMSE values are acquired for test images Tripoli-1 and Tripoli-2 when α+β=2 or α+β=3. In this study, refining parameters α and β are restricted with α+β=3.

**Figure 13:**
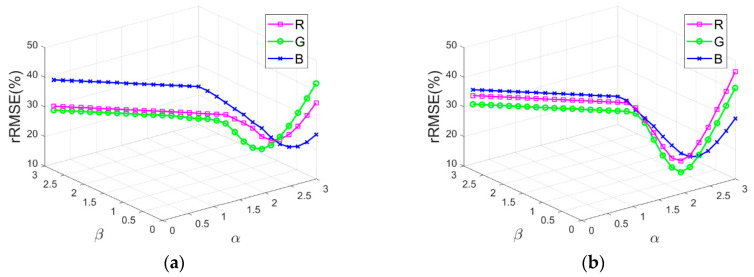
rRMSE of shadow compensation results with various α and β values for test images. (**a**) Tripoli-1. (**b**) Tripoli-2.

Additionally, the sensitivity of refining parameters α and β are further analyzed by valuing α and β from 0.1 to 3 with an interval of 0.1 in the additional experiments with test images Tripoli-1 and Tripoli-2. Figure 13a,b depict rRMSE of shadow compensation results with various α and β values for test images Tripoli-1 and Tripoli-2 in terms of R, G, and B components, respectively. As presented in Figure 13a,b, lower rRMSE values are acquired for test images Tripoli-1 and Tripoli-2 in terms of R, G, and B components, when parameters (α, β) are set as (2.4, 0.6), (2.5, 0.5), (2.6, 0.4), (2.7, 0.3), or (2.8, 0.2), which shows that better shadow compensation results are achieved with these parameter values of (α, β). Hence, in this study, we develop the IRB shadow compensation approach over test images with α=2.6 and β=0.4. 

### 4.4. Shadow Difference Analysis 

**Figure 14:**
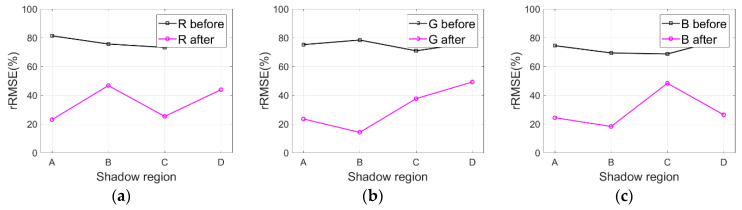
rRMSE of various shadow areas of images compensated before and after by the IRB approach for the test image Tripoli-1 in terms of R, G, and B components. (**a**) R. (**b**) G. (**c**) B.

**Figure 15:**
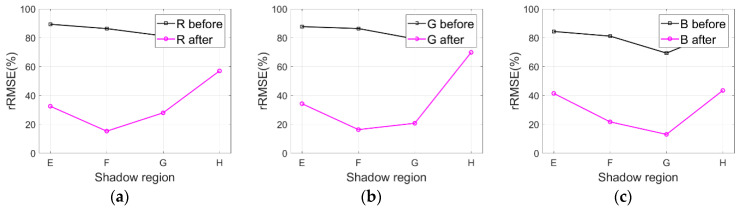
rRMSE of various shadow areas of images compensated before and after by the IRB approach for the test image Tripoli-2 in terms of R, G, and B components. (**a**) R. (**b**) G. (**c**) B.

In order to discuss shadow difference between different shadow areas in the same image, we particularly labelled several shadow regions with different shadows and analyzed the compensation results with rRMSE for test images Tripoli-1 and Tripoli-2. Specifically, as shown in Figure 3a, regions A-D are labelled in the test image Tripoli-1 (i.e., small buildings and grass in region A, blue houses in region B, and nearby shadow of buildings in regions C and D). Similarly, regions E-H are also labelled in the test image Tripoli-2 shown in Figure 3b (i.e., grass under building shadow in region E, asphalt roads in region F, tops of buildings in region G, and grass under small shadow in region H). Figure 14a–c and Figure 15a–c respectively depict the rRMSE of various shadow areas of images compensated before and after by the IRB approach for test images Tripoli-1 and Tripoli-2 in terms of R, G, and B components. 

As shown in Figure 14a–c and Figure 15a–c, smaller rRMSE values of various shadow areas of the resulting images are acquired for test images Tripoli-1 and Tripoli-2 in terms of R, G, and B components compared with the corresponding original ones, which reveals the fact that the IRB approach can well compensate feature information of shadow regions. As for the distinction of the rRMSE value for different shadow regions (like regions A-D in the test image Tripoli-1 and regions E-H in the test image Tripoli-2), the reflectivity of different ground features is the main reason. Therefore, external work will be carried out in the future for exploring the reflectivity of typical ground features. 

### 4.5. IRB Method Generalization Analysis 

**Figure 16:**
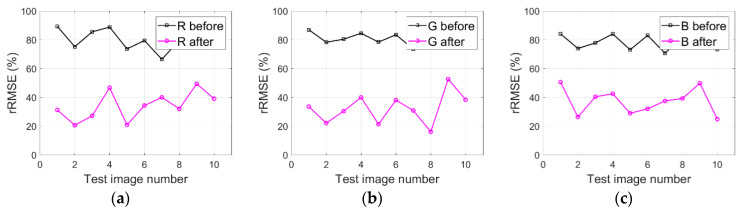
IRB method generalization analysis for WV3-Tripoli images in terms of R, G, and B components. (**a**) R. (**b**) G. (**c**) B.

**Figure 17:**
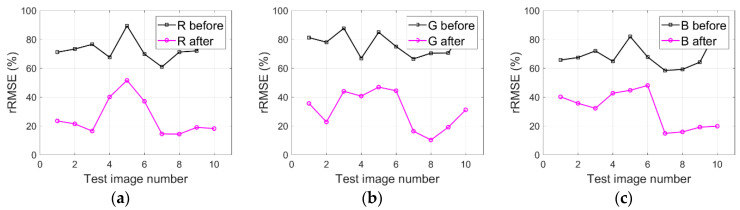
IRB method generalization analysis for WV3-Rio images in terms of R, G, and B components. (**a**) R. (**b**) G. (**c**) B.

**Figure 18:**
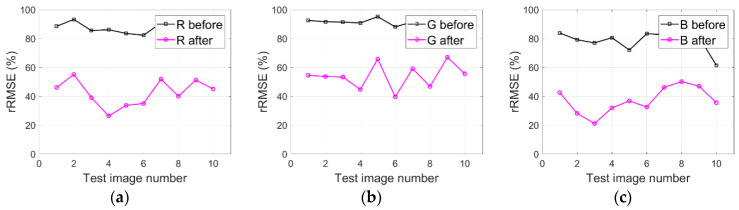
IRB method generalization analysis for WV2-WDC images in terms of R, G, and B components. (**a**) R. (**b**) G. (**c**) B.

As described in Section 2, the IRB approach is evaluated with many images (i.e., WV3-Tripoli, WV3-Rio, and WV2-WDC) to validate the IRB method generalization. Figure 16a–c respectively illustrate rRMSE values of both the original WV3-Tripoli images and the shadow compensated WV3-Tripoli images in terms of R, G, and B components. Similarly, Figure 17a–c and Figure 18a–c also depict the corresponding rRMSE values for WV3-Rio and WV2-WDC images compensated before and after by the IRB approach in terms of R, G, and B components. 

As can be observed in Figure 16a–c, smaller rRMSE values are achieved by the resulting WV3-Tripoli images in terms of R, G, and B components compared with the original WV3-Tripoli images. Figure 17a–c and Figure 18a–c also show similar phenomena for WV3-Rio and WV2-WDC images, which together deliver the IRB approach and is able to well compensate shadow information for HSR multispectral satellite remote sensing images. Provided this situation, two test images of WV3-Tripoli are particularly selected in this paper to assessing the IRB approach both qualitatively and quantitively against several shadow compensation methods (i.e., LCC, LSCR, MSR, HF, and DELM), as previously described in Section 2. 

## 5. Conclusions

In this paper, we developed and validated a simple but effective shadow compensation approach for high spatial resolution optical multispectral satellite remote sensing images on the basis of the satellite sensor imaging mechanism and radiative transfer theory. According to the distinct surface irradiance difference between non-shadow and nearby shadow areas, an assumption is first arranged for shadow areas under which shadow areas acquire the absent direct irradiance compared to the nearby non-shadow areas containing the same type features. Then, the solution for shadow compensation is further developed by estimating the path radiance and computing irradiance coefficient. Finally, refining operations are applied to optimize the initial shadow compensation solution. Additionally, comparative experiments are carried out to validate the proposed irradiance restoration based (IRB) shadow compensation. Shadow compensation performance of the proposed IRB method is assessed by both qualitative visual sense comparison and quantitative analysis against shadow compensation results by other comparative shadow compensation methods (i.e., LCC, LSCR, MSR, HF, and DELM). The shadow compensation results and the corresponding small rRMSE of the resulting images together reveal that the proposed IRB approach not only preserves information of features in non-shadow areas, but also performs well in restoring information of objects in shadow regions. Additionally, the proposed IRB method is more reasonable because it is developed on the basis of the satellite sensor imaging mechanism and radiative transfer theory, rather than only image processing (like LCC, MSR, and HF). The result images are more suitable for the subsequent image interpretation of high-resolution remote sensing images (such as change detection and surface feature reflectivity reversion). In the future, we will attempt to improve shadow compensation performance by further settling shadow detection problems and considering more influential details for the path radiance estimation combined with our current study. 

## Figures and Tables

**Table 1:** Eight multispectral bands of the WorldView-3 remote sensing image [41].

Band Number	Band Name	Wavelength Range (nm)
B1	Coastal	397–454
B2	Blue	445–517
B3	Green	507–586
B4	Yellow	580–629
B5	Red	626–696
B6	Red edge	698–749
B7	Near-IR1	765–899
B8	Near-IR2	857–1039

**Table 2:** Five relative scattering models [47].

Atmospheric Conditions	Relative Scattering Model
Very clear	$\lambda^{-4}$
Clear	$\lambda^{-2}$
Moderate	$\lambda^{-1}$
Hazy	$\lambda^{-0.7}$
Very hazy	$\lambda^{-0.5}$

## References

[1] Marcello J., Medina A., Eugenio F. (2013). Evaluation of spatial and spectral effectiveness of pixel-level fusion techniques. IEEE Geosci. Remote Sens. Lett..

[2] Fauvel M., Chanussot J., Benediktsson J.A. (2012). A spatial–spectral kernel-based approach for the classification of remote-sensing images. Pattern Recognit..

[3] Eugenio F., Marcello J., Martin J. (2015). High-resolution maps of Bathymetry and Benthic habitats in shallow-water environments using multispectral remote sensing imagery. IEEE Trans. Geosci. Remote Sens..

[4] Marcello J., Eugenio F., Perdomo U., Medina A. (2016). Assessment of atmospheric algorithms to retrieve vegetation in natural protected areas using multispectral high resolution imagery. Sensors.

[5] Martin J., Eugenio F., Marcello J., Medina A. (2016). Automatic Sun glint removal of multispectral high-resolution Worldview-2 imagery for retrieving coastal shallow water parameters. Remote Sens..

[6] Zhao J., Zhong Y., Shu H., Zhang L. (2016). High-resolution image classification integrating spectral-spatial-location cues by conditional random fields. IEEE Trans. Image Process..

[7] Huang S., Miao Y., Yuan F., Gnyp M.L., Yao Y., Cao Q., Wang H., Lenz-Wiedemann V.I.S., Bareth G. (2017). Potential of RapidEye and WorldView-2 satellite data for improving rice nitrogen status monitoring at different growth stages. Remote Sens..

[8] Lan T., Xue X., Li J., Han C., Long K. (2017). A high-dynamic-range optical remote sensing imaging method for digital TDI CMOS. Appl. Sci..

[9] Wen Z.F., Shao G.F., Mirza Z.A., Chen J.L., Lü M.Q., Wu S.J. (2015). Restoration of shadows in multispectral imagery using surface reflectance relationships with nearby similar areas. Int. J. Remote Sens..

[10] Ma L., Jiang B.T., Jiang X.W., Tian Y. Shadow removal in remote sensing images using features sample matting. Proceedings of the 2015 IEEE International Geoscience and Remote Sensing Symposium.

[11] Silva G.F., Carneiro G.B., Doth R., Amaral L.A., Azevedo D.F.G.d. (2017). Near real-time shadow detection and removal in aerial motion imagery application. J. Photogramm. Remote Sens..

[12] Tsai V.J.D. Automated shadow compensation in color aerial images. Proceedings of the 2006 Annual Conference of the American Society for Photogrammetry and Remote Sensing.

[13] Tsai V.J.D. (2006). A comparative study on shadow compensation of color aerial images in invariant color models. IEEE Trans. Geosci. Remote Sens..

[14] Yamazaki F., Liu W., Takasaki M. Charactersitics of shadow and removal of its effects for remote sensing imagery. Proceedings of the 2009 IEEE International Geoscience and Remote Sensing Symposium.

[15] Guo J.H., Liang L., Gong P. (2010). Removing shadows from Google Earth images. Int. J. Remote Sens..

[16] Liu W., Yamazaki F. (2012). Object-based shadow extraction and correction of high-resolution optical satellite images. IEEE J. Sel. Top. Appl. Earth Obs. Remote Sens..

[17] Zigh E., Belbachir M.F., Kadiri M., Djebbouri M., Kouninef B. (2015). New shadow detection and removal approach to improve neural stereo correspondence of dense urban VHR remote sensing images. Eur. J. Remote Sens..

[18] Anoopa S., Dhanya V., Jubilant J.K. (2016). Shadow detection and removal using tri-class based thresholding and shadow matting technique. Procedia Technol..

[19] Su N., Zhang Y., Tian S., Yan Y.M., Miao X.Y. (2016). Shadow detection and removal for occluded object information recovery in urban high-resolution panchromatic satellite images. IEEE J. Sel. Top. Appl. Earth Obs. Remote Sens..

[20] Wang Q.J., Tian Q.J., Lin Q.Z., Li M.X., Wang L.M. An improved algorithm for shadow restoration of high spatial resolution imagery. Proceedings of the Remote Sensing of the Environment: 16th National Symposium on Remote Sensing of China.

[21] Mostafa Y. (2017). A review on various shadow detection and compensation techniques in remote sensing images. Can. J. Remote Sens..

[22] Wu S.-T., Hsieh Y.-T., Chen C.-T., Chen J.-C. (2014). A comparison of 4 shadow compensation techniques for land cover classification of shaded areas from high radiometric resolution aerial images. Can. J. Remote Sens..

[23] Zhang H.Y., Sun K.M., Li W.Z. (2014). Object-oriented shadow detection and removal from urban high-resolution remote sensing images. IEEE Trans. Geosci. Remote Sens..

[24] Wang S.G., Wang Y. Shadow detection and compensation in high resolution satellite image based on Retinex. Proceedings of the 5th International Conference on Image and Graphics.

[25] Hao N., Liao H. (2010). Homomorphic filtering based shadow removing method for high resolution remote sensing images. Softw. Guide.

[26] Suzuki A., Shio A., Ami H., Ohtsuka S. Dynamic shadow compensation of aerial images based on color and spatial analysis. Proceedings of the 15th International Conference on Pattern Recognition.

[27] Wang S.G., Guo Z.J., Li D.R. (2003). Shadow detection and compensation for color aerial images. Geo-Spat. Inf..

[28] Guo J., Tian Q. (2004). A shadow detection and compensation method for IKONOS imagery. Remote Sens. Inf..

[29] Sarabandi P., Yamazaki F., Matsuoka M., Kiremidjian A. Shadow detection and radiometric restoration in satellite high resolution images. Proceedings of the 2004 IEEE International Geoscience and Remote Sensing Symposium.

[30] Su J., Lin X.G., Liu D.Z. An automatic shadow detection and compensation method for remote sensed color images. Proceedings of the 8th International Conference on Signal Processing.

[31] Chen Y., Wen D., Jing L., Shi P. (2007). Shadow information recovery in urban areas from very high resolution satellite imagery. Int. J. Remote Sens..

[32] Xiao Z., Huang J. (2004). Shadow eliminating using edge fuzzied retinex in urban colored aerial image. Chin. J. Stereol. Image Anal..

[33] Ye Q., Xie H., Xu Q. Removing shadows from high-resolution urban aerial images based on color constancy. Proceedings of the International Archives of the Photogrammetry, Remote Sensing and Spatial Information Sciences.

[34] Finlayson G.D., Trezzi E. Shades of gray and color constancy. Proceedings of the 12th Color Imaging Conference: Color Science and Engineering Systems, Technologies, Applications.

[35] Lin Z., Ren C., Yao N., Xie F. (2013). A shadow compensation method for aerial image. Geomat. Inf. Sci. Wuhan Univ..

[36] Chen S., Zou L. Chest radiographic image enhancement based on multi-scale Retinex technique. Proceedings of the 3rd International Conference on Bioinformatics and Biomedical Engineering.

[37] Guo R.Q., Dai Q.Y., Hoiem D. (2013). Paired regions for shadow detection and removal. IEEE Trans. Pattern Anal. Mach. Intell..

[38] Zheng Q., Wang Q. (2008). A shadow reconstruction method for QuickBird satellite remote sensing imagery. Comput. Eng. Appl..

[39] Bai T., Jin W. (2006). Priciple and Technology of Electronic Imaging.

[40] Richards J.A. (2013). Remote Sensing Digital Image Analysis.

[41] DG2017_WorldView-3_DS. https://dg-cms-uploads-production.s3.amazon-aws.com/uploads/document/file/95/DG2017_WorldView-3_DS.pdf.

[42] Mostafa Y., Abdelhafiz A. (2017). Accurate shadow detection from high-resolution satellite images. IEEE Geosci. Remote Sens. Lett..

[43] Wang Q.J., Yan L., Yuan Q.Q., Ma Z.L. (2017). An atomatic shadow detection method for VHR remote sensing orthoimagery. Remote Sens..

[44] Mo N., Zhu R.X., Yan L., Zhao Z. (2018). Deshadowing of urban airborne imagery based on object-oriented automatic shadow detection and regional matching compensation. IEEE J. Sel. Top. Appl. Earth Obs. Remote Sens..

[45] Vicente T.F.Y., Hoai M., Samaras D. (2018). Leave-one-out kernel optimization for shadow detection and removal. IEEE Trans. Pattern Anal. Mach. Intell..

[46] Han H., Han C., Lan T., Huang L., Hu C., Xue X. (2020). Automatic shadow detection for multispectral satellite remote sensing images in invariant color spaces. Appl. Sci..

[47] Chavez P.S. (1988). An improved dark-object subtraction technique for atmospheric scattering correction of multispectral data. Remote Sens. Environ..

[48] Chavez P.S. (1996). Image-based atmospheric corrections-revisited and improved. Photogramm. Eng. Remote Sens..

[49] Zhang X., Chen F., He H. (2016). Shadow detection in high resolution remote sensing images using multiple features. ACA Autom. Sin..

